# Analysis of Shear Performance of Multi-Bolt Shear Connectors

**DOI:** 10.3390/ma16031032

**Published:** 2023-01-23

**Authors:** Rongtian Xie, Tao Yang, Baojun Li, Shiyuan Liu, Yongbing Zhang

**Affiliations:** 1 College of Civil Engineering and Architecture, Guangxi University, Nanning 530004, China; 2 Key Laboratory of Disaster Prevention and Structural Safety of Ministry of Education, Nanning 530004, China; 3 Guangxi Key Laboratory of Disaster Prevention and Engineering Safety, Nanning 530004, China; 4 Guangxi Transportation Research Institute, Nanning 530007, China

**Keywords:** multi-bolt shear connector, shear resistance, numerical analysis, load–slip relationship

## Abstract

Bolt shear connectors used in prefabricated steel–concrete composite beams can be arranged into a group to enhance the construction efficiency, which will cause the multi-bolt effect and further affect the shear performance of bolt connectors. This paper developed three-dimensional finite element models of push-out specimens to investigate the shear performance of multi-bolt connectors. Numerical results showed that the friction coefficient at the interfaces between the steel girders and precast concrete (PC) slabs and bolt preload dramatically improved the initial stiffness of bolts; when the longitudinal spacing of bolts was reduced from 100 mm to 60 mm, the decrease in the average peak load per bolt was 3.5%, 9.2%, and 11.4% for bolts of 16 mm, 20 mm, and 24 mm diameters. A modified calculation method for the shear resistance of multi-bolt shear connectors was proposed based on the numerical analysis, and a simplified model of shear load versus relative slip was further developed.

## 1. Introduction

Steel–concrete composite beams are widely used in building structures due to the ample use of the properties of the individual materials compared to traditional bare steel and concrete beams [1,2]. In such composite beams, the composite action degree and synergistic bending capacity between the precast concrete (PC) slab and steel beam are dependent on shear connectors, allowing each component of the composite structure to be fully employed [3,4,5]. Studs were the most widely used shear connectors in the past. With the development of the assembly design of composite beams, bolt shear connectors are gradually utilized as an alternative to studs to facilitate the construction and demolition of composite structures. Therefore, the shear performance of bolt connectors has been broadly investigated in recent years. Ataei et al. [6,7] proved that the bolt diameter and the tensile strength of bolts are the main parameters affecting the shear behaviors of demountable bolt connectors. Some researchers [8,9,10] experimentally validated that the demountable shear connectors exhibited similar load-bearing capacities to head studs, except for ductility. The demountable shear connectors presented a higher ductility than head studs in some cases [11]. Meanwhile, the assessment methods for assessing the load-bearing capacity of bolt shear connectors have been presented [12,13]. As a result, bolt shear connectors are practicable to be used in prefabricated composite structures.

Several bolt shear connectors can be arranged as a group to enhance construction efficiency. Related research results show that the average ultimate shear resistance of bolt connectors arranged in a group is usually smaller than that of a single bolt connector [14,15]. In addition, the adverse of the multi-bolt effect on the shear resistance of multi-bolt shear connectors has been proved by many researchers [16,17]. Therefore, the shear behaviors of multi-bolt shear connectors are worthy of further investigation. This paper aims to evaluate the effect of common design parameters on the shear behaviors of multi-bolt shear connectors in the finite element (FE) methods, and the shear resistance and inherent changing rule of shear stiffness of multi-bolt connectors are also to be investigated.

## 2. Overview of the Push-Out Tests of Multi-Bolt Connectors

**Figure 1:**
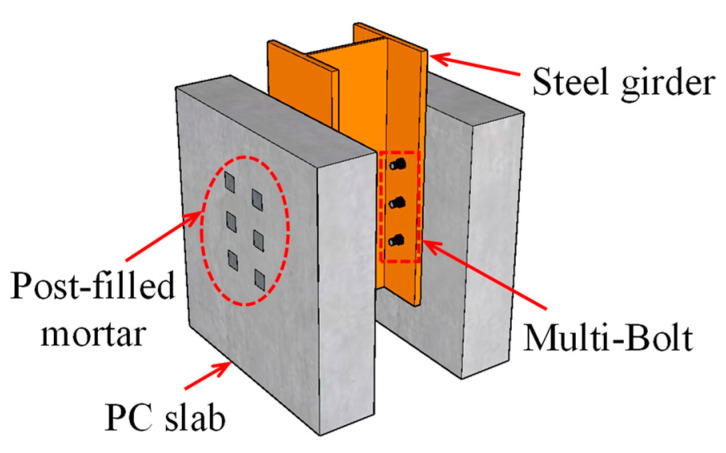
Schematic diagram of the push-out specimen (e.g., TS4).

**Figure 2:**
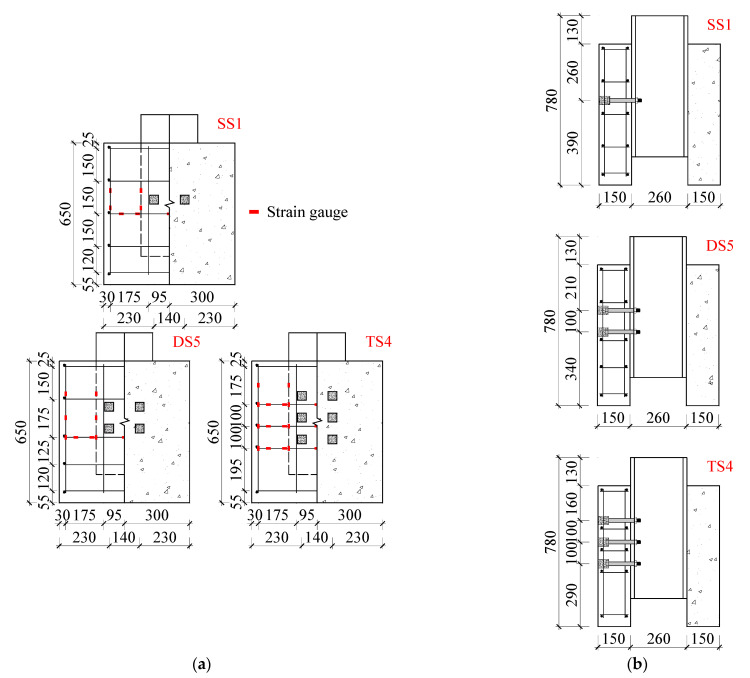
Dimensions and reinforcement details: (**a**) elevation view; (**b**) side view.

Yang et al. [18] reported the push-out tests on multi-bolt connectors, which were designed following Eurocode 4 [19]. The push-out specimens named SS1, DS5, and TS4 were selected as the reference specimens in the following numerical studies, which had single, double, and triple rows of bolts arranged in each PC slab, respectively. Figure 1 shows the schematic diagram of the push-out specimens, taking TS4 as an example. Figure 2 shows the dimensions and reinforcement details of the assembled push-out specimens. The welded H-shaped steel girder, with a cross-section of 260 mm × 260 mm × 18 mm × 10 mm (flange width, section height, flange thickness, and web thickness), was manufactured with steel plates by manual arc welding. The length, width, and depth of the PC slabs were 650 mm, 600 mm, and 150 mm, respectively. The diameter of the preserved holds in the PC slabs was 17.5 mm, the end of which was enlarged to 40 mm × 40 mm × 50 mm (length, width, depth) cuboid holes to contain the bolt heads. Grade 8.8 M16 (16 mm in diameter) high-strength bolts were adopted as the shear connectors to connect the PC slabs with the steel girders, to which a preload of 30 kN was applied after assembly. Then, the enlarged holes in the PC slabs were post-filled with high-strength mortar. As the interface between the steel girder and the PC slabs for each specimen was lubricated by lithium-based grease, the reported experimental results can only reflect the contribution of the bolts themselves to the shear resistance, and that of the preload of bolts is not included.

The mechanical properties of the steel girder and slab reinforcement were tested by the standard tensile coupons. The manufacturing methods of the coupons and the testing process can be referred to as the China code GB/T 228-2010 [20]. The principal parameters of the steel members are listed in Table 1. Meanwhile, the prismatic and cube concrete specimens were made simultaneously as the PC slabs were cast, which were cured in the same outdoor condition for 28 days. The prismatic concrete specimens (100 mm width, 100 mm length, and 300 mm height) were used to test Young’s modulus and the axial compressive strength of the concrete; the tensile strength of the concrete was tested by the cube concrete specimens with the side length of 150 mm. All the tests of the concrete were performed following the China code GB/T 50081-2019 [21], and the measured mechanical properties of the concrete are listed in Table 2. Additionally, the compressive strength of the post-filled mortar was 78.5 MPa, which was obtained by testing on three cube blocks with side lengths of 70.7 mm following China code JGJ/T 70-2009 [22]. According to the factory inspector’s report, the ultimate tensile strength of the M16 bolt was 880 MPa.

**Figure 3:**
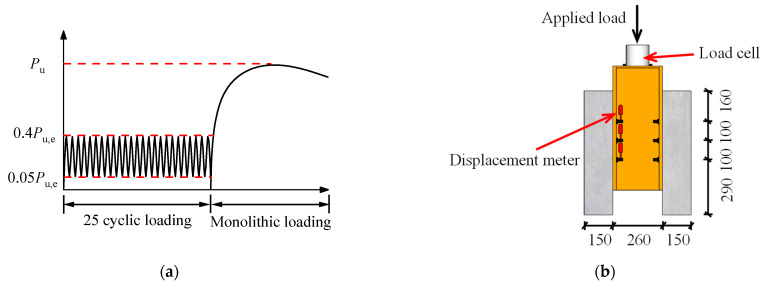
Loading regime and test setup: (**a**) loading regime; (**b**) test setup.

Figure 3 shows the setup of the push-out tests. A vertical load was applied to the push-out specimens by a servo-hydraulic actuator, and the loading regime complied with Eurocode 4 [19]. Figure 3a depicts the loading procedure, which was divided into two steps: (1) The specimens were loaded for 25 cycles within the range of 0.05*P*_u,e_ to 0.4*P*_u,e_, where *P*_u,e_ represents the estimated ultimate shear resistance calculated following Eurocode 4. (2) The monolithic load was applied to the specimens under displacement control at a rate of 0.3 mm/min. The final failure of the specimens was identified when the load-carrying capacity dropped to 0.8*P*_u_, where *P*_u_ represents the actual peak load of the specimens. Displacement meters were set at the interfaces corresponding to the bolt connectors to monitor the relative slip between the steel girder and the PC slabs, as shown in Figure 3b. Strain gauges were mounted on the slab reinforcement to record the strain development, as shown in Figure 2a.

## 3. Finite Element (FE) Modeling Method

### 3.1. Geometry, Element Type, and Mesh

**Figure 4:**
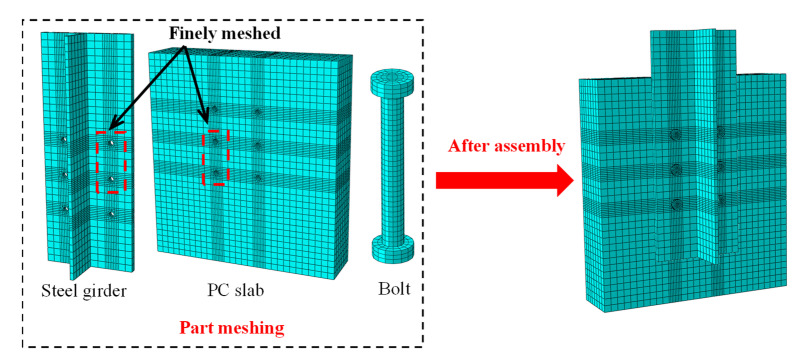
Mesh details of the FE models.

Three-dimensional FE models were built in the software ABAQUS to evaluate the shear performance of multi-bolt shear connectors referring to the aforementioned three push-out specimens. Only half of each model was simulated, considering the symmetry of the specimens and the boundary conditions. Figure 4 shows the mesh details of the FE models. Three-dimensional two-node truss elements (T3D2) were used to model the slab reinforcement. In addition, the other components, including the PC slab, the bolts, and the steel girder, were simulated by the three-dimensional eight-node solid elements (C3D8R). The mesh size of the bolts was taken at 4.0 mm, and that of the other components was 20 mm. Note that the fine mesh approach, i.e., the number of seeds near the bolts was adjusted from two to four, is adopted in the regions around the bolts to achieve accurate results.

### 3.2. Interaction, Loading, and Boundary Conditions

**Figure 5:**
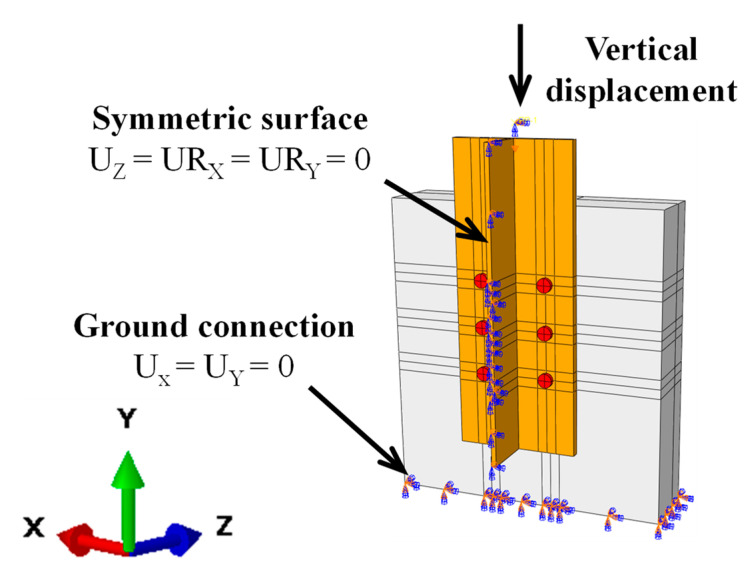
Boundary conditions.

The surface-to-surface contact (Standard) provided by ABAQUS was applied at all the interfaces among the bolts, the PC slab, and the steel girder. The contact properties were defined in normal and tangential directions separately. The “hard contact” algorithm is used to depict the contacted behavior in the normal direction, which allows interaction forces to be transferred without intrusion. The “penalty” algorithm was adopted to describe the tangential responses, which could simulate the effect of friction by introducing a friction coefficient. The friction coefficient at the interfaces between the PC slabs and steel girder was set as zero due to the lithium-based grease interface treatment in the tests. Then, the friction coefficient was set as 0.3 for the steel-to-steel interfaces and 0.45 for the steel-to-concrete interfaces when the interfacial friction had to be considered. The interaction between the post-filled mortal and PC slab was described by the “tie” model without considering the relative slip. In addition, the reinforcing bars were embedded into the PC slabs, with the interface slip being neglected. The boundary conditions of the models are shown in Figure 5. The loading procedure can be divided into two-step: (1) preload the bolt by activating the “bolt load” option; (2) apply an anticipated vertical displacement on the loading points till the final failure of FE models occurs.

### 3.3. Mechanical Properties of Concrete and Steel

The bilinear model was used to describe the stress–strain relationship of steel [23]. In addition, the material characteristics of concrete were simulated by the concrete damaged plasticity model (CDPM). The plasticity parameters of concrete in the CDPM are listed in Table 3. The specific mechanical indicators of the concrete and steel were mentioned in Section 2. The concrete stress–strain responses under compression and tension provided by the China code [24] are used as the calculation model to import into ABAQUS in the CDPM for simulation.

### 3.4. Verification of FE Results

**Figure 6:**
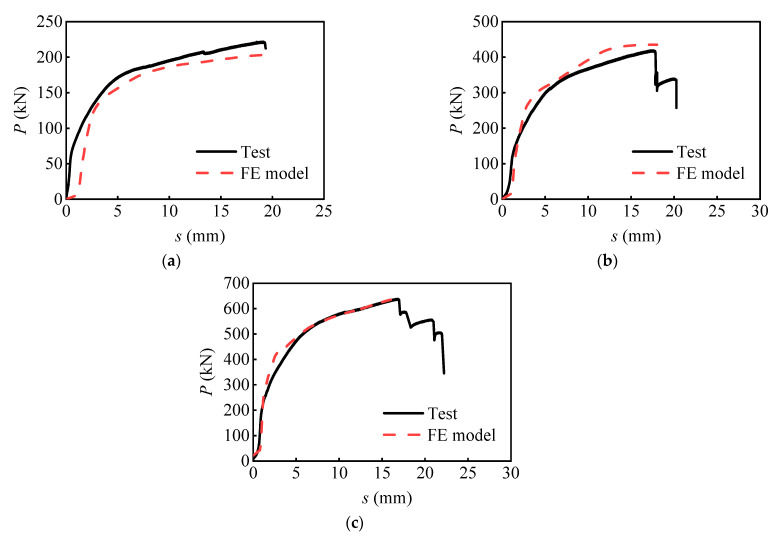
Comparison of the numerical and experimental results: (**a**) SS1; (**b**) DS5; (**c**) TS4.

**Figure 7:**
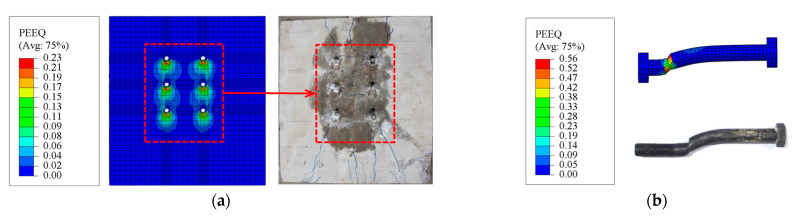
Comparison of the failure modes (e.g., TS4): (**a**) damage of the PC slab; (**b**) deformation of the bolt shear connector.

The local and global behaviors of the developed FE models were compared to the experiment results to guarantee the reliability of the modeling method. It is regarded that the final failure occurred when the interface slip reached 1.0 times the bolt diameter. Figure 6 shows the comparison of the load versus relative slip curves between the numerical and experimental results. It can be seen that the curves obtained from the numerical analysis exhibit a similar variation trend to that of the tests. The maximum error between the peak load obtained from the numerical analyses and the experiments was only 8.7%. The discrepancy between the load versus slip curves of SS1 in the initial loading stage could be attributed to the bolt-hole clearance. Moreover, Figure 7 shows the failure mode of the PC slab and bolts estimated by equivalent plastic strain (PEEQ), which agrees well with the experimental observations. Therefore, the FE model can reproduce the global and local behaviors of the push-out specimens of bolt shear connectors.

## 4. Parametric Analysis and Discussion

The parametric analysis was carried out in the developed modeling method to investigate pertinent variables on the shear performance of multi-bolt shear connectors. A total of 29 FE models were built, referring to the above modeling procedure. Table 4 presents the design parameters of the models, where *P*_i_ is the bolt preload; *μ*_i_ is the interface friction coefficient between the steel girder and the PC slab; *ρ*_t_ is the transverse reinforcement ratio; *d*_bolt_ is the bolt diameter; *n*_row_ is the row numbers of bolts; *l*_s_ is the longitudinal bolt spacing; *d*_s_ is the depth of the PC slab; *d*_hole_ is the diameter of the preformed hole in the PC slab.

### 4.1. Shear Performance of Individual-Bolt in the Multi-Bolt Connectors

**Figure 8:**
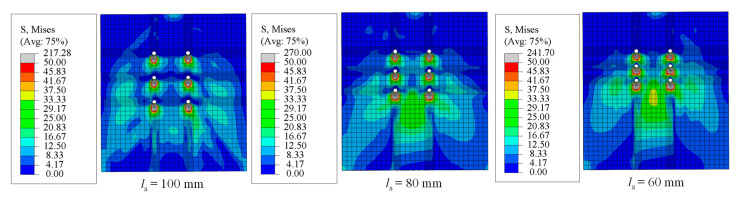
Distribution of von Mises stress on the PC slab.

**Figure 9:**
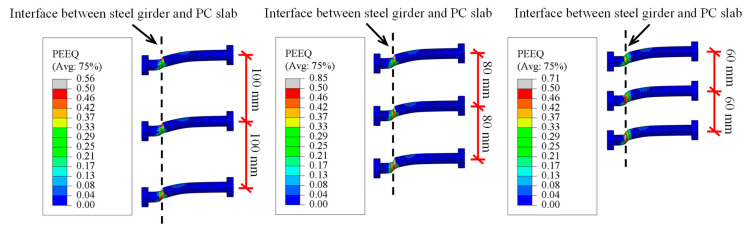
The deformation of an individual bolt in the multi-bolt shear connectors.

Three FE models with a bolt diameter of 16 mm and different longitudinal bolt spacing (*l*_s_) of 100 mm, 80 mm, and 60 mm are set as a contrast group to evaluate the effect of surrounding bolts on the shear performance of the individual bolt. Figure 8 compares the distribution of von Mises stress on the PC slab at the final failure. While *l*_s_ = 100 mm, the distribution of von Mises stress around each preserved hole is independent. The border of the nephogram overlaps with each other when *l*_s_ decreases. The upper bolts affected the stress conditions of the lower bolts, which caused deterioration of the stress status of the PC slab near the lower bolt. Figure 9 compares the shear deformation of an individual bolt in a bolt group at the final failure. Similarly, the bolt below shows more significant deformation according to the PEEQ nephogram. Therefore, the decrease in the longitudinal bolt spacing is adverse to the shear performance of the individual bolt in the multi-bolt connectors, especially for the bolts below.

### 4.2. Effect of Design Parameters on the Shear Performance of Bolt Connectors

#### 4.2.1. Concrete Compressive Strength

**Figure 10:**
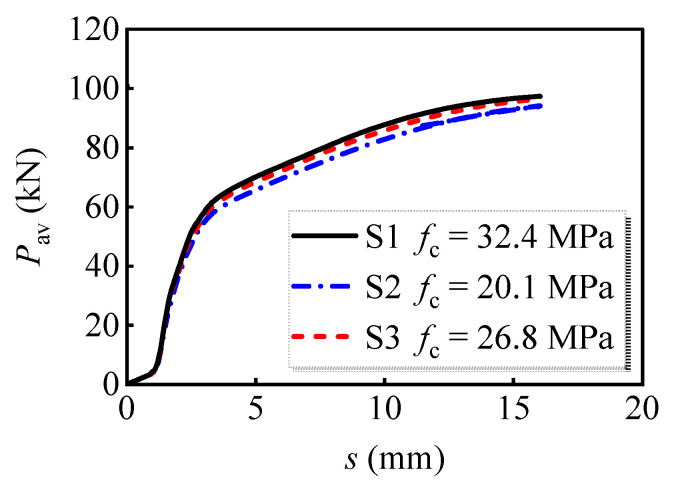
Average load versus slip curves for models with different concrete compressive strength.

Figure 10 illustrates the effect of the concrete compressive strength on the load versus slip curves, where *P*_av_ represents the average shear resistance per bolt. The ultimate shear resistance increased by 3.4% as the compressive strength increased from 20.1 MPa to 32.4 MPa. Therefore, the shear performance of multi-bolt shear connectors slightly decreased with the decrease in concrete compressive strength.

#### 4.2.2. Bolt Preload and Friction Coefficient

**Figure 11:**
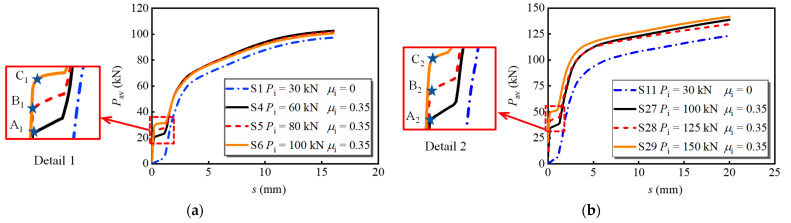
Average load versus slip curves for models with different bolt preloads: (**a**) *d*_bolt_ = 16 mm; (**b**) *d*_bolt_ = 20 mm.

**Figure 12:**
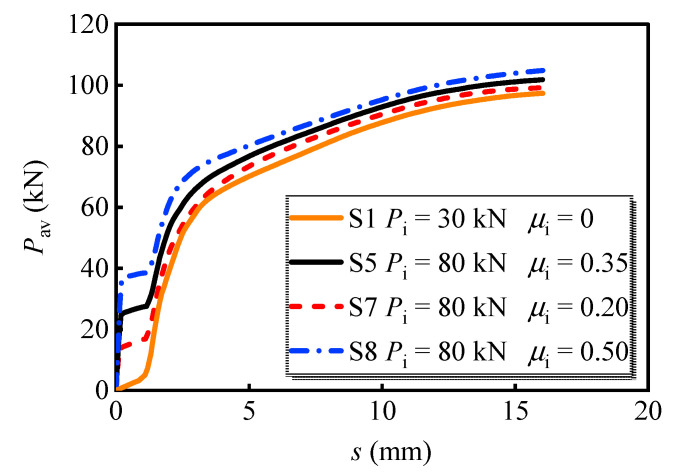
Average load versus slip curves for models with different friction coefficients.

Figure 11 shows the load versus slip curves of the models with bolt diameters of 16 mm and 20 mm under various preloads. The friction coefficient between the steel girder and the PC slab interface was set as 0.35. Points A-C marked in details 1 and 2 correspond to the moment that the interface friction of each specimen was overcome. The following conclusions can be drawn:

(1) For bolts with a diameter of 16 mm (see Figure 11a), the interface slip quickly increases with the applied load at the initial loading stage when the interface friction coefficient is zero. Once the interface friction is considered, the shear force is first borne by the interface friction between the steel girder and the PC slab. The load corresponding to point A_1_ is 20.5 kN, which is 17.6% and 31.7% lower than those at points B_1_ and C_1_, respectively. The ultimate load-bearing capacity of S4-S6 is at least 5.2% greater than S1. Figure 11b shows the curves of bolt connectors of 20 mm diameter, which possess a similar developing tendency to that of bolts with a diameter of 16 mm. The applied load corresponding to A_2_ is 34.3 kN, which is 16.7% and 30.7% lower than those at points B_2_ and C_2_, respectively. The ultimate load-bearing capacity of S27-S29 is at least 9.0% greater than S11. Therefore, it can be concluded that the application of preload dramatically increases the initial stiffness of the bolt and benefits the ultimate load-bearing capacity. A comparison shows that the increase in the ultimate load-bearing capacity is more evident for the bolt with a greater diameter.
(2) The effects of the steel–concrete interface friction coefficient are also investigated. S1, S5, S7, and S8 were set as a group, which processes the bolt preload of 80 kN and various interface friction coefficients between the steel girder and PC slab. The average load versus slip curves is shown in Figure 12. Clearly, the increase in friction coefficient advanced the load-bearing capacity of the multi-bolt shear connector. Meanwhile, the load-bearing capacity at the moment that the interface was overcome increased by 160.2% when the friction coefficient increased from 0.2 to 0.5. In addition, the increase in friction coefficient causes a 5.6% increase in the ultimate load-bearing capacity. Therefore, properly enhancing the interface friction coefficient is beneficial for the load-bearing capacity of the bolts.

#### 4.2.3. Transverse Reinforcement Ratio

**Figure 13:**
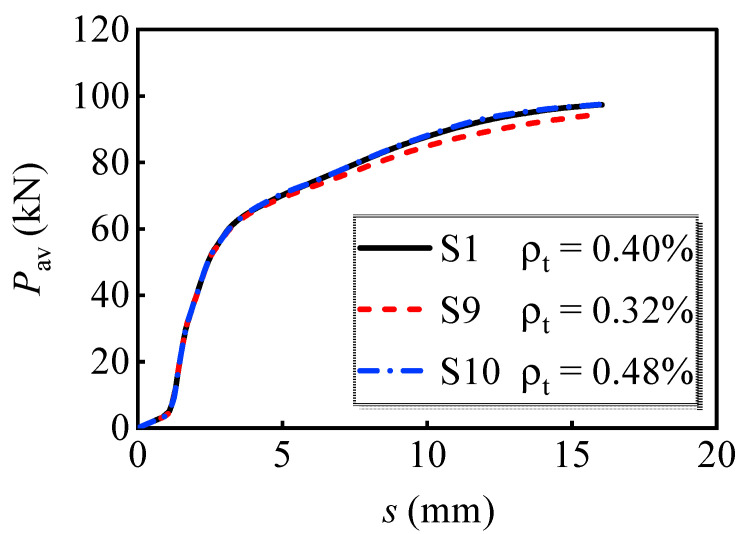
Variation of the load versus slip curves for models with different transverse reinforcement ratios.

Figure 13 shows the average load versus slip curves with different transverse reinforcement ratios. The average ultimate load-bearing capacity of the individual bolt increased by 3.2% when the transverse reinforcement ratios increased from 0.32% to 0.48%. These curves almost overlap, except for S9, which has a slightly lower load-bearing capacity in the latter loading stage. Therefore, the transverse reinforcement ratio has little effect on the shear resistance of multi-bolt shear connectors.

#### 4.2.4. Bolt Diameter

**Figure 14:**
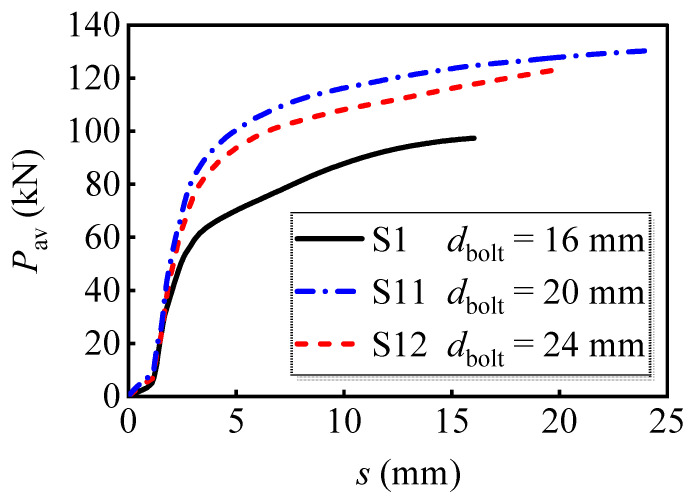
Average load versus slip curves of models with different bolt diameters.

Numerical analysis was conducted for models with different bolt diameters of 16 mm, 20 mm, and 24 mm, respectively. The applied load versus slip curves is shown in Figure 14. The ultimate load-bearing capacity per bolt increased by 26.7% when the bolt diameter increased from 16 mm to 20 mm; however, it only increased by 5.9% as the diameter increased from 20 mm to 24 mm. The comparison indicates that the increase in the bolt diameter significantly enhances the load-bearing capacity of bolts. In addition, this amplification becomes inconspicuous as the bolt diameter gradually increases. This may be owing to the increase in bolt diameter amplifying the discrepancy in stiffness between bolts and PC slabs. In this case, the PC slab could not provide effective restraint for the bolts with greater diameter.

#### 4.2.5. Longitudinal Bolt Spacing

**Figure 15:**
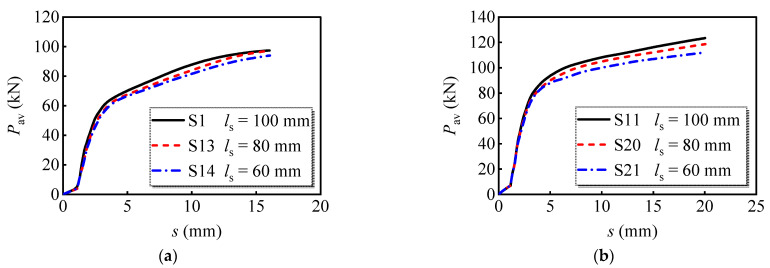

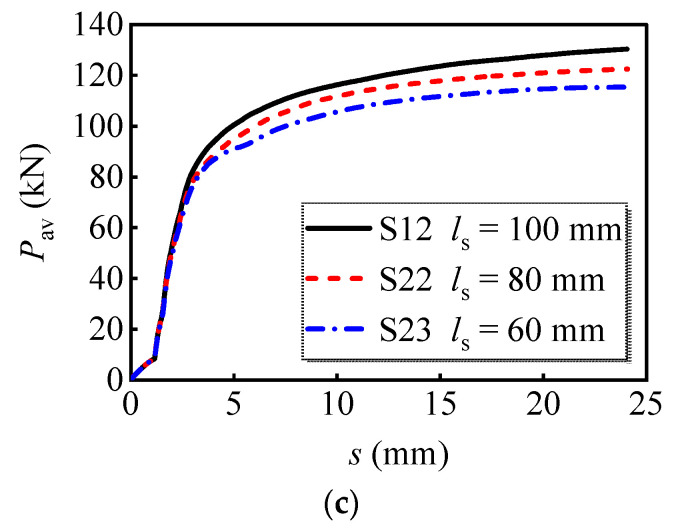
Average load versus slip curves of models with different longitudinal bolt spacing: (**a**) *d*_bolt_ = 16 mm; (**b**) *d*_bolt_ = 20 mm; (**c**) *d*_bolt_ = 24 mm.

Three groups of FE models with bolt diameters of 16 mm, 20 mm, and 24 mm, respectively, were developed to evaluate the effects of longitudinal bolt spacing on the shear performance of multi-bolt connectors. Figure 15 compares the load versus slip curves. It can be found that changing the longitudinal bolt spacing mainly affects the load-bearing capacity. When the longitudinal spacing was reduced from 100 mm to 60 mm, the reduction in peak load per bolt was 3.5%, 9.2%, and 11.4% for bolts with 16 mm, 20 mm, and 24 mm diameters, respectively. Accordingly, the decrease in longitudinal bolt spacing reduces the load-bearing capacity of the multi-bolt shear connector, which is more evident for bolts with greater diameters.

#### 4.2.6. Row Number of Bolts

**Figure 16:**
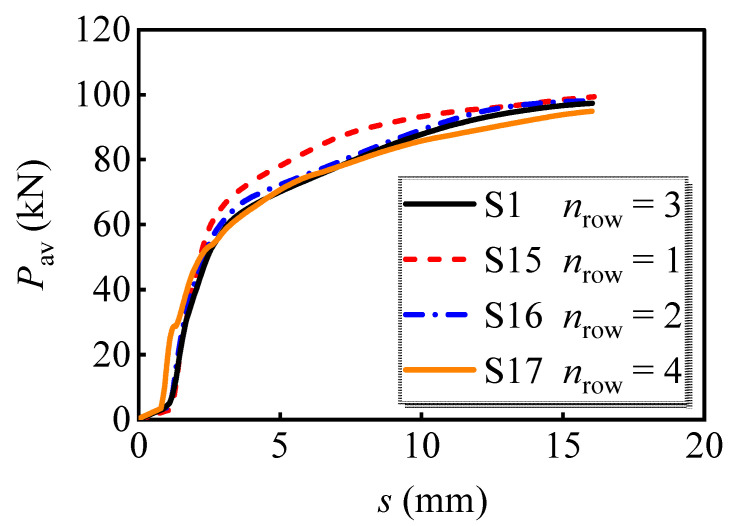
Average load versus slip curves for models with different row numbers of bolts.

The analyzed results on the load versus slip curves of the models with the different row numbers of bolts are displayed in Figure 16. It can be seen that the model with a single row of bolts processes the greatest load-bearing capacity. The ultimate load-bearing capacity of S15 is 1.2%, 2.1%, and 4.7% greater than S16, S1, and S17, respectively. This phenomenon can be attributed to the stress status of an individual bolt being deteriorated by the surrounding bolts, referring to the von Mises stress nephogram shown in Section 4.1. Therefore, the individual bolt in the multi-bolt shear connectors bears an uneven shear force. The bolt bearing the maximum shear force would rupture first and then cause the successive failure of the remaining bolts.

#### 4.2.7. Depth of the PC Slab

**Figure 17:**
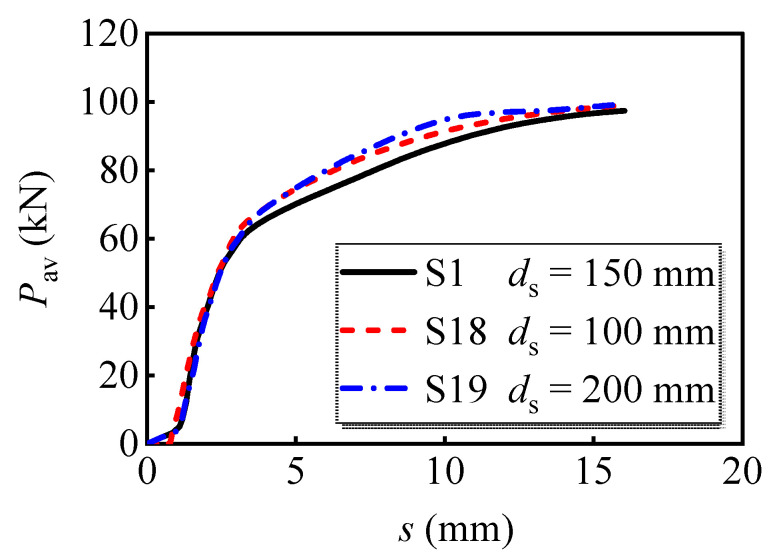
Average load versus slip curves for models with different slab depths.

Figure 17 shows the load versus slip curves with different depths of the PC slab. The analyzed results show that the model with a greater slab depth processes better load-bearing capacity in the latter loading stage. Meanwhile, the ultimate load-bearing capacity per bolt of S19 is 0.5% and 2.0% greater than S1 and S18, respectively, indicating that the slab depth adjustment has no significant effects on the ultimate load-bearing capacity of bolt shear connectors.

#### 4.2.8. Clearance between Bolt and Bolt Hole

**Figure 18:**
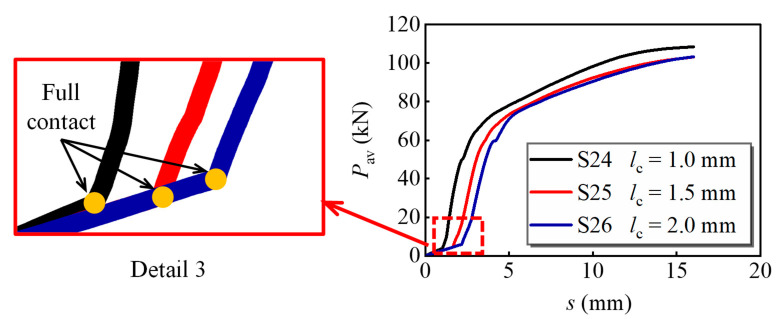
Variation of the load versus slip curves for models with different bolt-hole clearances.

Figure 18 depicts the applied load versus slip curves with different bolt-hole clearances, where *l*_c_ = (*d*_hole_ − *d*_bolt_)/2 represents the bolt-hole clearance. The ultimate load-bearing capacity of S24 is 5.0% greater than that of S26, which indicates that the increase in *l*_c_ decreased the average load-bearing capacity per bolt. The relative slip corresponding to the full contact of bolt and bolt hole for S24-S26 was 1.01 mm, 1.56 mm, and 2.04 mm, respectively, which is almost the same as *l*_c_, as shown in detail 3. Therefore, the contact between the bolt and the hole wall is highly relevant to bolt-hole clearance.

## 5. Assessment of Ultimate Shear Resistance for Bolt Shear Connectors

Based on push-out tests, Yang et al. [18] have proposed a calculation model to predict the ultimate shear resistance of M16 bolts arranged in a group, and the calculation model can be described as follows:

(1) $$N_{u}=0.6\alpha A_{\mathrm{sc}}f_{u}$$

where *A*_sc_ represents the effective shear area of the shear connectors; *f*_u_ represents the ultimate tensile strength of the bolts. The coefficient *α* is taken as 0.9 for the bolts arranged as a group in three rows, and the coefficient is taken as 1.0 for the bolts arranged uniformly or as a group in double rows.

**Figure 19:**
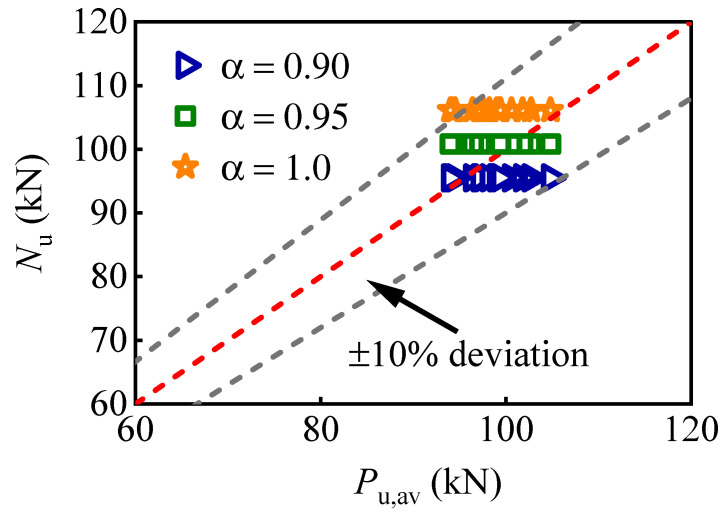
Comparison of the average ultimate shear resistance based on the calculation and FE results.

This model considers the multiple bolts effect and is suitable for predicting the ultimate shear resistance of M16 bolts arranged in a group without interface friction. However, the influence of bolt preload and interface friction is not considered in this calculation model. Therefore, it is recommended to introduce another coefficient *α* = 0.95 to estimate the ultimate shear resistance of multi-bolt connectors, in which the effect of friction is considered. Figure 19 shows the comparison between the calculation and FE results, where *P*_u,av_ represents the average ultimate shear resistance per bolt obtained from numerical analysis, and *N*_u_ represents the calculated ultimate shear resistance per bolt following Equation (1).

The statistical indicators of different coefficient values of Equation (1) are listed in Table 5. The subscripts represent the value of the coefficient. The comparison shows that after modifying the coefficient value as 0.95, the calculation model processes the smallest average, variance, and coefficient of variation. Therefore, adjusting the value of the coefficient *α* to 0.95 is a minor and effective adjustment, which can predict the shear strength of bolt shear connectors with reasonable accuracy and is suitable for use in the design of multi-bolt shear connectors.

## 6. Shear Force Versus Slip Relationship of Multi-Bolt Shear Connectors

**Figure 20:**
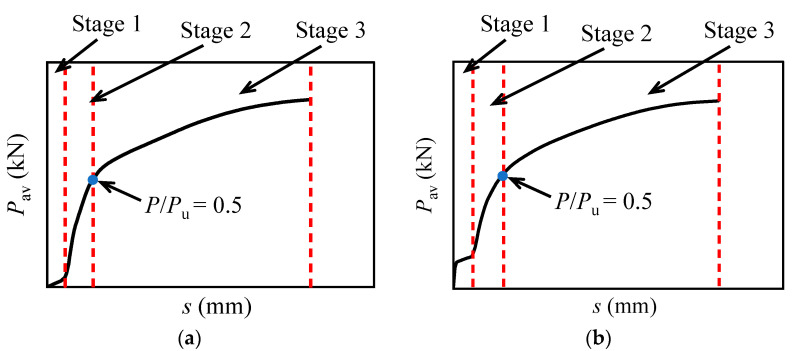
Schematic diagram of the mechanical behavior: (**a**) without preload and interface friction; (**b**) with preload and interface friction.

The shear force versus relative slip relationship is crucial in evaluating the load-bearing capacity and deformability of the composite beams. Three-stage characteristics for the load versus slip relationship for the M16 bolts in multi-bolt connectors can be concluded according to the numerical results. Stage 1: The interface slip increases rapidly and linearly with the applied load due to the bolt-hole clearance, as shown in Figure 20a. This stage ends when the bolts contact the wall of the preserved hole. Once the interfacial friction is considered, the applied load is to be resisted by the friction first, and slip appears after the friction is overcome, as shown in Figure 20b. Stage 2: the bolts resist the shear force, and the tangent stiffness of the bolt shear connectors almost keeps constant and becomes greater than that in stage 1. Stage 3: after the *P*/*P*_u_ exceeds 0.5, the stiffness of the bolt shear connectors degenerates with the load until the final failure occurs. Hence, the shear force versus relative slip relationship can be divided into two categories, as shown in Figure 20.

Accordingly, two simplified calculation models of the shear load versus slip relationship for M16 bolt shear connectors were developed. The model for bolts without considering the interface friction and preload can be obtained through Equation (2):

(2) $$\frac{N}{N_{u}}=\left\{\begin{array}{ll} 0.042s & s\leq l_{c}\\ 0.042l_{c}+\frac{s-l_{c}}{2.18+0.92(s-l_{c})} & l_{c}<s<16\;\mathrm{mm} \end{array}\right.$$

The model considering the interface friction and preload can be depicted by Equation (3):

(3) $$\frac{N}{N_{u}}=\left\{\begin{array}{ll} 0.93P_{i}\mu_{i}\times 10^{-5}+0.028s & s\leq l_{c}\\ 0.93P_{i}\mu_{i}\times 10^{-5}+0.028l_{c}+\xi \frac{s-l_{c}+44.01}{4.13+3.08(s-l_{c})} & l_{c}<s<16\;\mathrm{mm} \end{array}\right.$$

where *ξ* represents the effects of the bolt preload and the interface friction on the load-bearing capacity of the bolt, and the unit of *P*_i_ is Newtons (N). The coefficient *ξ* can be described as Equation (4):

(4) $$\xi =\frac{1.67(s-l_{c})}{4.59P_{i}\mu_{i}\times 10^{-4}+26.94}$$

**Figure 21:**
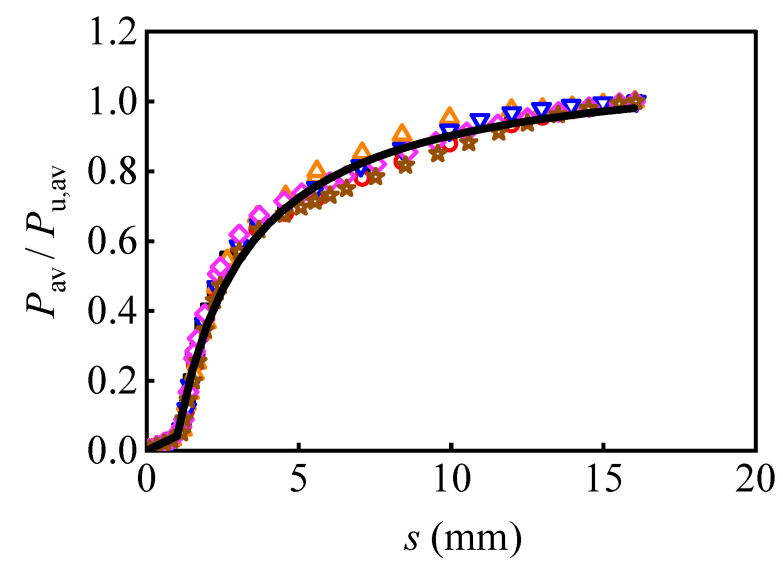
The regression curve without considering preload and interface friction.

**Figure 22:**
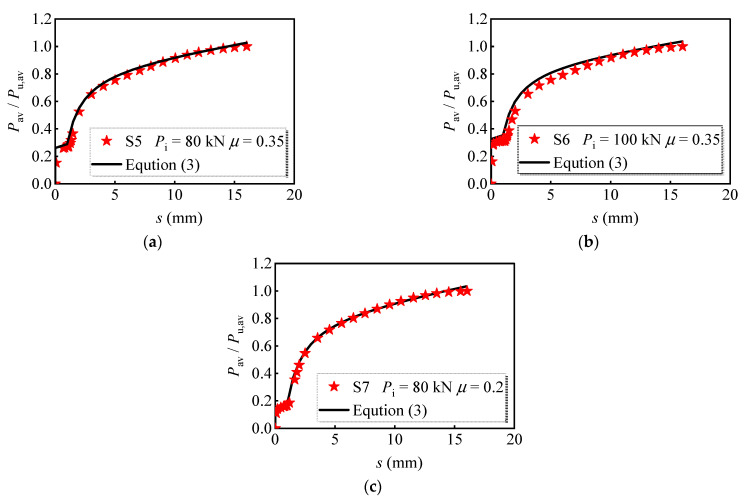
The regression curve considering preload and interface friction: (**a**) S5; (**b**) S6; (**c**) S7.

Figure 21 compares the developed theoretical model, Equation (2), with the representative values selected from the numerical results. The R-squared for Equation (2) was 0.990, and the fitted curve agrees well with the numerical results. Meanwhile, the theoretical model, Equation (3), is also compared with the numerical results, as shown in Figure 22. The curves and the selected models meet well with each other, and the R-squared for Equation (3) was 0.991. Therefore, it can be concluded that the developed theoretical model can be employed in predicting the shear load versus relative slip curves for the M16 bolt in multi-bolt shear connectors.

## 7. Conclusions

Three-dimensional finite models were developed to investigate the shear performance of the multi-bolt shear connector. The accuracy of the developed model was validated against the available test results. Parametric analyses were performed to study the influence of extensive parameters, such as the bolt preload and the bolt diameter, on the shear performance of the multi-bolt shear connector. The following conclusions can be drawn based on the investigation:

(1) The concrete strength, transverse reinforcement ratio, and depth of the slab had a negligible effect on the shear resistance of multi-bolt shear connectors. The bolt preload, the friction coefficient of the interface between the steel girder and PC slab, bolt diameter, and longitudinal bolt spacing are the primary parameters that affect the shear performance of the multi-bolt shear connectors.
(2) The bolt preload and the friction coefficient of the interface between the steel girder and PC slab dramatically improved the initial shear stiffness of the bolts. The friction at the steel–concrete interface first resists the applied shear force, and the interface relative slip is negligible before the interface friction is overcome. Moreover, the increase in the bolt preload and the interface friction coefficient are beneficial for the ultimate load-bearing capacity of the bolts, which is more evident for the bolts with a greater diameter.
(3) When the longitudinal bolt spacing was reduced from 100 mm to 60 mm, the decrease in average peak load per bolt was 3.5%, 9.2%, and 11.4% for multi-bolts with diameters of 16 mm, 20 mm, and 24 mm. Therefore, the decrease in longitudinal bolt spacing reduces the load-bearing capacity of the bolts, which becomes more evident for bolts with greater diameters.
(4) The increase in the bolt diameter significantly enhances the load-bearing capacity of the bolt. The ultimate load-bearing capacity of the bolt increased by 26.7% when the bolt diameter increased from 16 mm to 20 mm; however, it only increased by 5.9% as the diameter increased from 20 mm to 24 mm. The increase in bolt diameter amplifies the discrepancy in stiffness between bolts and PC slabs; hence, the PC slab could not provide effective restraint for the bolts while greater diameter bolts were used.
(5) A calculation model was proposed and verified to exhibit reasonable accuracy in predicting the shear resistance of multi-bolt shear connectors. Meanwhile, two simplified shear force versus slip models for multi-bolt shear connectors with a diameter of 16 mm were developed, which could be used for the prediction of shear stiffness of multi-bolt connectors considering the interface friction and bolt preload influence.

## Figures and Tables

**Table 1:** Mechanical properties of steel.

Items	Thickness/Diameter (mm)	Yield Strength (MPa)	Tensile Strength (MPa)	Young’s Modulus (GPa)
Steel beam flange	18	343.5	483.0	210.0
Steel beam web	10	300.0	435.0	204.0
Reinforcing bars	10	424.3	682.0	182.0

**Table 2:** Mechanical properties of concrete.

Axial Compressive Strength *f*_c_ (MPa)	Tensile Strength *f*_t_ (MPa)	Young’s Modulus *E*_c_ (GPa)
37.3	3.4	37.8

**Table 3:** Plasticity parameters for CDPM.

Dilation Angle	Eccentricity	Stress Ratio	Shape Factor	Viscosity Factor
30°	0.1	1.16	0.6667	0.001

**Table 4:** Parametric design of FE models.

ID	*f*_c_ (MPa)	*P*_i_ (kN)	*μ*_i_	*ρ*_t_ (%)	*d*_bolt_ (mm)	*n*_row_	*l*_s_ (mm)	*d*_s_ (mm)	*d*_hole_ (mm)
S1	32.4	30.0	0	0.40	16	3	100	150	18
S2	20.1	30.0	0	0.40	16	3	100	150	18
S3	26.8	30.0	0	0.40	16	3	100	150	18
S4	32.4	60.0	0.35	0.40	16	3	100	150	18
S5	32.4	80.0	0.35	0.40	16	3	100	150	18
S6	32.4	100.0	0.35	0.40	16	3	100	150	18
S7	32.4	80.0	0.2	0.40	16	3	100	150	18
S8	32.4	80.0	0.5	0.40	16	3	100	150	18
S9	32.4	30.0	0	0.32	16	3	100	150	18
S10	32.4	30.0	0	0.48	16	3	100	150	18
S11	32.4	30.0	0	0.40	20	3	100	150	18
S12	32.4	30.0	0	0.40	24	3	100	150	18
S13	32.4	30.0	0	0.40	16	3	80	150	18
S14	32.4	30.0	0	0.40	16	3	60	150	18
S15	32.4	30.0	0	0.40	16	1	N.A.	150	18
S16	32.4	30.0	0	0.40	16	2	100	150	18
S17	32.4	30.0	0	0.40	16	4	100	100	18
S18	32.4	30.0	0	0.40	16	3	100	100	18
S19	32.4	30.0	0	0.40	16	3	100	200	18
S20	32.4	30.0	0	0.40	20	3	80	150	18
S21	32.4	30.0	0	0.40	20	3	60	150	18
S22	32.4	30.0	0	0.40	24	3	80	150	18
S23	32.4	30.0	0	0.40	24	3	60	150	18
S24	32.4	30.0	0	0.40	16	3	100	150	18
S25	32.4	30.0	0	0.40	16	3	100	150	19
S26	32.4	30.0	0	0.40	16	3	100	150	20
S27	32.4	100.0	0.35	0.40	20	3	100	150	18
S28	32.4	125.0	0.35	0.40	20	3	100	150	18
S29	32.4	150.0	0.35	0.40	20	3	100	150	18

**Table 5:** Statistic indicators of different coefficient values.

Statistical Indicators	*N_u,_*_[*α = 0.90*]_/*P*_u,av_	*N_u,_*_[*α = 0.95*]_/*P*_u,av_	*N_u,_*_[*α = 1.0*]_/*P*_u,av_
Average	0.970	1.024	1.078
Variance	0.0017	0.0015	0.0071
Coefficient of variation	0.0427	0.0378	0.0782

## Data Availability

Not applicable.
